# MetaboAnalyst 3.0—making metabolomics more meaningful

**DOI:** 10.1093/nar/gkv380

**Published:** 2015-04-20

**Authors:** Jianguo Xia, Igor V. Sinelnikov, Beomsoo Han, David S. Wishart

**Affiliations:** 1 Institute of Parasitology, and Department of Animal Science, McGill University, Ste. Ann de Bellevue, QC H9X 3V9, Canada; 2 Department of Microbiology and Immunology, McGill University, Montreal, QC H3A 2B4, Canada; 3 Department of Computing Science, University of Alberta, Edmonton, AB T6G 2E8, Canada; 4 Department of Biological Sciences, University of Alberta, Edmonton, AB T6G 2E9, Canada; 5 National Institute for Nanotechnology, 11421 Saskatchewan Drive, Edmonton, AB T6G 2M9, Canada

## Abstract

MetaboAnalyst (www.metaboanalyst.ca) is a web server designed to permit comprehensive metabolomic data analysis, visualization and interpretation. It supports a wide range of complex statistical calculations and high quality graphical rendering functions that require significant computational resources. First introduced in 2009, MetaboAnalyst has experienced more than a 50X growth in user traffic (>50 000 jobs processed each month). In order to keep up with the rapidly increasing computational demands and a growing number of requests to support translational and systems biology applications, we performed a substantial rewrite and major feature upgrade of the server. The result is MetaboAnalyst 3.0. By completely re-implementing the MetaboAnalyst suite using the latest web framework technologies, we have been able substantially improve its performance, capacity and user interactivity. Three new modules have also been added including: (i) a module for biomarker analysis based on the calculation of receiver operating characteristic curves; (ii) a module for sample size estimation and power analysis for improved planning of metabolomics studies and (iii) a module to support integrative pathway analysis for both genes and metabolites. In addition, popular features found in existing modules have been significantly enhanced by upgrading the graphical output, expanding the compound libraries and by adding support for more diverse organisms.

## INTRODUCTION

MetaboAnalyst is a widely used, web-based system that supports comprehensive metabolomic data analysis, visualization and interpretation. The first release of MetaboAnalyst (introduced in 2009) contained just a single module focusing on metabolomic data processing and statistical analysis (1). The second version of MetaboAnalyst (released in 2012) contained four functional modules that supported expanded capabilities in metabolomic functional analysis and data interpretation (2). Since its introduction, MetaboAnalyst 2.0 has been continuously updated by improving existing functions, adding minor new features, upgrading the underlying design framework as well as the server hardware. These enhancements made the server increasingly popular within the metabolomics community. Indeed, the number of data analysis jobs submitted to the server has grown from ∼800/month (in 2010) to ∼3200/month (in 2013) to a current ∼40 000/month (in 2014). At the same time there has also been a significant shift in the type and complexity of metabolomics studies that are being routinely performed. In particular, the last several years have seen an increasing number of metabolomics-based biomarker studies in agricultural, biomedical and clinical settings (3–10). Additionally there are now growing numbers of complex, multi-omic studies being performed that integrate metabolomics data with genomics, epigenomics or proteomics data over large populations (11–15). As a consequence, user-friendly tools to support biomarker analysis, population-based experimental design and multi-omic data integration have been among the most requested features by MetaboAnalyst's users. Likewise, with continuing advances in web-based technologies such as HTML5 and AJAX (asynchronous JavaScript and XML), the demands for more interactive visualization tools and larger-scale data visualizations have never been stronger.

Given the demand for new functions to support emerging applications in metabolomics, the need for a more computationally efficient implementation to accommodate the tremendous growth in jobs submitted to MetaboAnalyst, and the growing expectation for more powerful data visualization features we decided to undertake a near complete rewrite of MetaboAnalyst. Hence, we developed MetaboAnalyst 3.0. This new version represents a substantially upgraded and a significantly improved offering over MetaboAnalyst 2.0. The main features in MetaboAnalyst 3.0 include:

1. A completely re-implemented web framework based on the latest web technologies for significantly improved speed, performance and user interactivity;
2. A consolidated interface with substantially improved graphical outputs for MetaboAnalyst's most popular analyses along with new features for better interactivity and customization;
3. Substantial updates to MetaboAnalyst's compound library and metabolic pathways library based on the latest versions of HMDB (16), SMPDB (17) and KEGG (18);
4. A new module for biomarker analysis featuring tools to perform receiver operating characteristic (ROC) curve analyses using single or multiple metabolites;
5. A new module to support sample size estimation and power analysis for designing population-based or clinical metabolomic studies;
6. A new module for integrated pathway analysis for combining results from transcriptomic and metabolomic studies

### General design of MetaboAnalyst 3.0

During the re-implementation of MetaboAnalyst, we made every effort to maintain the same ‘look and feel’ of the earlier versions in order to reduce the learning curve for current users. We only introduced interface changes if they led to significant performance gains, if they were more intuitive to use, or if they offered extra functionalities. Perhaps the most obvious change is the appearance of eight independent modules when users start a MetaboAnalyst session. MetaboAnalyst 2.0 originally offered four functional modules that shared the same navigation tree, allowing users to traverse to different modules during a given session. However, this design required the server to load all analysis modules, which resulted in large memory footprint. With the 50X increase in user traffic and server workload, this large memory consumption became increasingly burdensome, leading to a substantial slow down in performance. In version 3.0, each module is now an independent component with its own navigation tree. The new design not only reduces memory usage but also simplifies the navigation panel making it less prone to operational errors. It also makes it more straightforward to add new modules to future versions of MetaboAnalyst.

The eight functional modules in MetaboAnalyst 3.0 can be grouped into three general categories—Category 1: exploratory statistical analysis (*Statistical Analysis* and *Time Series Analysis*); Category 2: functional analysis (*Enrichment Analysis, Pathway Analysis* and *Integrated Pathway Analysis*) and Category 3: advanced methods for translational studies (*Biomarker Analysis* and *Power Analysis*). In addition, there is also an *Other Utilities* module currently containing a compound ID conversion tool and a specialized function for lipidomic data analysis (19).

**Figure 1.**
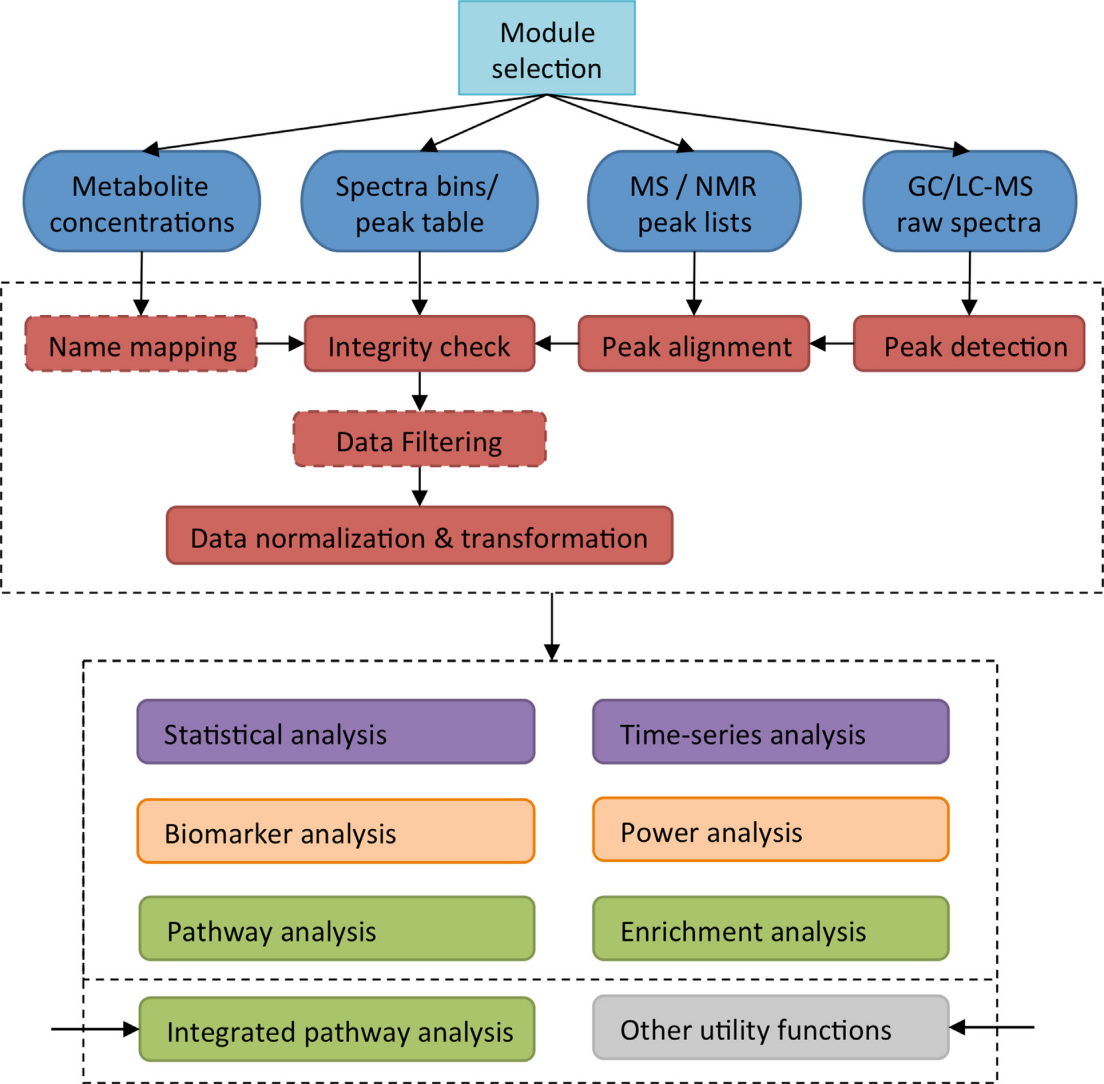
MetaboAnalyst 3.0 Flowchart. This figure illustrates the general logic and data processing pipeline behind MetaboAnalyst. Different functions will be applied to process different types of data into matrices. The red boxes with dashed boundaries indicate functions that are only triggered in certain data analysis scenarios. After data integrity checks and normalization steps have been completed, downstream statistical analyses (purple box), functional analyses (green box) or advanced analyses for translational studies (orange box) can be applied. Note that different inputs are required for integrated pathway analysis and for invoking some of the general utility functions.

A flow chart describing the overall design, structure and functional modules for MetaboAnalyst 3.0 is given in Figure 1. Depending on the selected module and the type of data uploaded, different processing methods will be applied to convert the data into a data table (or data matrix) with samples in rows and features (compounds, peaks or spectral bins) in columns. Advanced data processing steps such as missing value estimation and data filtering are also available. Most of the functions implemented in MetaboAnalyst 3.0's data processing steps have largely remained the same as in the previous version. Some small improvements have been made. For instance, a modern *Color Picker* is now available to allow users to freely select any color to label specific clusters or groups. The *Data Editor* now allows users to exclude samples, features or groups (for multi-group data only) during an analysis. In addition to the changes (both large and small) in MetaboAnalyst's data processing functions, there have also been a number of updates and additions to MetaboAnalyst's other functional modules. These changes are grouped according to their functional categories and described in further detail below.

### Category 1: exploratory statistical analysis

Besides completely re-engineering and consolidating MetaboAnalyst's user interface, significant effort was put into updating many of the exploratory statistical analysis elements in the *Statistical Analysis* and *Time Series Analysis* modules. The most significant enhancements have occurred with the scores/loadings plots, heatmaps and the feature details table.

**Figure 2.**
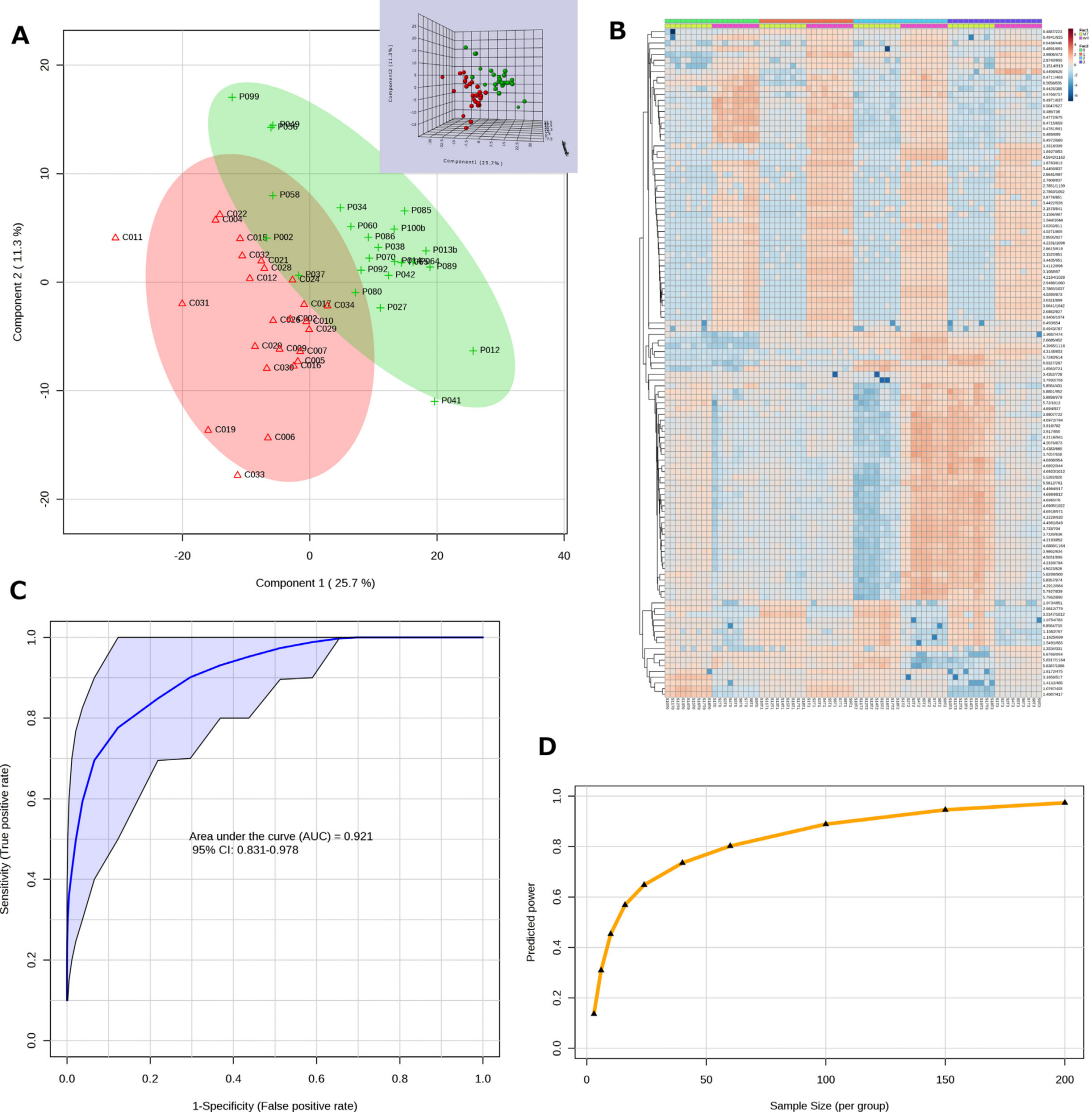
Sample screenshots from MetaboAnalyst 3.0. (**A**) A PLS-DA 2D scores plot with semi-transparent confidence intervals. The corresponding interactive 3D plot is shown in its top right corner. (**B**) Updated heatmaps automatically adjust their image size to ensure all data points are visible. (**C**) An ROC curve with a 95% confidence interval (marked in purple) generated from several manually selected biomarkers. (**D**) A summary plot showing the relationship between different sample sizes and predicted powers.

- *Scores/loadings plots*. The two common clustering and classification methods—principal component analysis (PCA) and partial least squares - discriminant analysis (PLS-DA) continue to be the most popular methods used in MetaboAnalyst. The most frequently viewed outputs from these two analyses are the scores and loadings plots. The scores plot provides an intuitive summary of the sample clustering patterns by projecting high-dimensional metabolomics data into two or three dimensions in a way that explains the maximal variance (PCA) or co-variance (PLS-DA) of the data; while the loading plot shows the underlying compounds responsible for such separation patterns. In developing MetaboAnalyst 3.0, we experimented with several different visualization approaches to improve both the quality and information content of the plots. In particular, the interface for two-dimensional (2D) scores plot now allows users to adjust a number of parameters to customize the graphical output. The corresponding loadings plot and the three-dimensional (3D) scores plot now support interactive visual exploration featuring point-and-click selection, zooming and rotating (3D only). An example graphical output is given in Figure 2A. These features run natively on all modern web browsers with JavaScript enabled. The 2D interactive visualization support has been extended to volcano plots as well as results from t-tests and ANOVA. The 3D feature has also been implemented for the interactive PCA (iPCA) method in the *Time-series Analysis* module.
- *Heatmaps*. Clustered heatmaps are another very popular visualization tool in MetaboAnalyst. Heatmaps allow users to easily visualize changing patterns in metabolite concentrations across samples and across experimental conditions. In contrast to the scores plots, heatmaps display the actual data values using carefully chosen color gradients. In the previous version of MetaboAnalyst, the heatmap was restricted to a fixed size, which led to nearly illegible graphs when very large datasets were being visualized. In MetaboAnalyst 3.0, we re-implemented the heatmap visualization tool using the R pheatmap package (version 0.7.7). In addition to the fixed size ‘Overview’, users can also choose the ‘Detail View’ which will automatically adjust the output image size based on the actual uploaded data size to ensure that the resulting heatmaps will be easily readable (up to 2000 features). This new implementation takes advantage of the auto-scroll capability of newer web framework components for displaying overflowing content without distorting the user interface. This enhancement has been applied to all heatmaps generated via hierarchical clustering, correlation analysis and two-factor clustering analysis in the *Time-series Analysis* module. An example heatmap is shown in Figure 2B.
- *Feature details table*. The feature details table is used to complement the graphical output from a standard statistical analysis by presenting the underlying numerical details through a hyperlink. It has been implemented for all methods that generate feature ranking results, such as t-tests, ANOVA, PCA/PLS-DA loadings, etc. Users can access the feature details table by clicking the table icon on the top-right corner of a corresponding image. To allow facile navigation of very large tables with thousands of features, we have re-implemented the underlying algorithms to be more computationally efficient and more interactive. These include new functions that allow flexible column sorting and name searching, in addition to the visualization of individual features.

### Category 2: functional analysis

There are two main approaches in functional analysis in MetaboAnalyst–metabolite set enrichment analysis (20) and metabolic pathway analysis (21). Both approaches work by comparing the significant compounds identified from the uploaded data to those pre-defined functional groups. The required inputs for these two analyses are metabolite concentration data or a list of metabolite names. Both inputs require a name-mapping step to standardize the compound IDs. In version 3.0, we have updated the underlying metabolite library based on the latest version of HMDB (16). This led to a 5X increase in the number of compounds or metabolite names available in MetaboAnalyst. We have also re-implemented the algorithm to improve the performance of fuzzy string matching, which has often been a significant computational bottleneck when the uploaded data contains many non-standard compound names. Based on user requests, we have also added support for more organisms for metabolic pathway analysis, such as *Schistosoma mansoni, Plasmodium falciparum, Trypanosoma brucei, Synechococcus elongatus*.

### New module #1: integrated pathway analysis

This is a new module introduced in MetaboAnalyst 3.0 to allow users to integrate data from two commonly performed omic experiments—gene expression experiments and metabolomics experiments. This approach exploits the models from KEGG metabolic pathways to complete the analysis. The underlying assumption behind this module is that by combining the evidence based on changes in both gene expression and metabolite concentrations, one is more likely to pinpoint the pathways involved in the underlying biological processes. To this end, users need to supply a list of genes and metabolites of interest that have been identified from the same samples or obtained under similar conditions. The metabolite list can be selected from the results of a previous analysis downloaded from MetaboAnalyst. Similarly, the gene list can be easily obtained using many excellent web-based tools such as GEPAS (22) or INVEX (23). After users have uploaded their data, the genes and metabolites are then mapped to KEGG metabolic pathways for over-representation analysis and pathway topology analysis (21). Topology analysis uses the structure of a given pathway to evaluate the relative importance of the genes/compounds based on their relative locations. Clicking on the name of a specific pathway will generate a graphical representation of that pathway highlighted with the matched genes/metabolites. Users need to keep in mind that unlike transcriptomics where the entire transcriptome is routinely mapped, current metabolomic technologies capture only a small portion of the metabolome. This difference can lead to potentially biased results. To address this issue, the current implementation of this omic integration module allows users to explore the enriched pathways based either on the joint evidence or on the evidence obtained from one particular omic platform for comparison.

### Advanced methods for translational or clinical studies

In recent years, there has been an increasing interest in the application of metabolomics for clinical or translational medicine studies such as biomarker identification for disease diagnosis, prognosis or monitoring. These studies often require specialized statistical analyses that are very different from the methods normally used in most metabolomic data analyses (which tend to focus on biological interpretation). In MetaboAnalyst 3.0, we have introduced two new modules to address these more pragmatic clinical or translational needs—the *Biomarker Analysis* module for biomarker identification and performance evaluation, and the *Power Analysis* module to support sample size estimation for clinical study design.

### New module #2: biomarker analysis

Biomarkers are objectively measurable biological characteristics that can be used to indicate certain conditions or disease states. The primary goal of biomarker analysis is to build a predictive model from one or more variables, which can be used to classify new subjects into specific groups (e.g. healthy versus diseased) with optimal sensitivity and specificity. Biological understanding is not a prerequisite for biomarker development. The procedures for biomarker analysis are formalized into three major steps: (i) biomarker selection, (ii) performance evaluation and (iii) model creation. Many different approaches are available for each step. For instance, the performance of a biomarker model can be assessed in several ways. The two most important performance measures are sensitivity (true positive rate) and specificity (true negative rate). For any test, there is usually a trade-off between these two measures. Choosing a different threshold may increase the sensitivity at the expense of lowering the specificity or vice versa. One of the best ways to observe how a decision threshold affects sensitivity and specificity is through an ROC curve. A ROC curve can be created by plotting the *sensitivity* against *1-specificity* at various threshold settings. It depicts the performance of a biomarker test over the complete range of possible decision boundaries. ROC curve analysis is widely considered to be the most objective and statistically valid method for biomarker performance evaluation. ROC curves are often summarized into a single metric known as the area under the ROC curve (AUC), which is widely used to compare the performance of different biomarker models. This new Biomarker Analysis module supports all common ROC-curve based biomarker analysis. These include:

- *Classical ROC curve analysis*. The section allows users to perform univariate ROC curve analysis for each compound. Users can generate ROC curves, to calculate the full AUC or partial AUC as well as their 95% confidence intervals, or to compute optimal cutoffs.
- *Multivariate ROC curve explorer*. This section allows users to explore the performance of different biomarker models automatically created through the built-in feature selection and performance evaluation procedures. Users can choose among three well-established multivariate algorithms including PLS-DA, support vector machines and random forests to perform ROC curve analyses.
- *ROC curve based model creation and evaluation*. This section allows users to manually select a subset of features and then test their performance using any of the three algorithms mentioned above. An example ROC curve output is shown in Figure 2C. This module also allows users to hold out a subset of samples for separate validation in addition to the built-in cross validation. The significance of the biomarker model can also be evaluated using permutation-based approaches.

Biomarker analysis often involves a number of very complex statistical procedures. More technical details about different algorithms are provided by the corresponding FAQs in MetaboAnalyst's online documentations. A more detailed introduction on how to use and interpret ROC curves within the context of metabolomics biomarker analysis can also be found in a recent comprehensive tutorial (24).

### New module #3: power analysis and sample size estimation

Statistical power is defined as the probability of detecting an effect, when the effect is present. For instance, let us assume a study that compares a specific effect or feature between a control population and a diseased population has a power of 0.8. Assuming we can conduct this study many times, then 80% of the time, we would get a statistically significant difference for that effect/feature between the two groups. The power of a study depends on three factors: (i) the magnitude of the effect in the population (effect size), (ii) the statistical significance criterion used in the test and (iii) the sample size used to detect the effect. In practice, the effect size can be estimated from a pilot data; the significance criterion (alpha level) is usually the *P* value in traditional univariate power analysis. Given these two constraints, researchers are usually most interested in knowing the sample size (number of subjects) required in order to obtain sufficient power in a given study.

However, estimating sample size for high-throughput omics studies is more complicated. Omics datasets are characterized by tens of thousands of features and a relatively small number of samples. Both the effect sizes and variances will have many values. A number of different methods have been proposed during the past years to deal with this issue (25–29). A general approach is that for high-dimensional omics data, the average power should be used instead of power, and significance levels need to take multiple testing into account using standard methods such as false discovery rate instead of raw *P* values.

The power analysis algorithm used in MetaboAnalyst 3.0 is based on the Bioconductor package SSPA as described by van Iterson *et al*. (26,29). This method uses the entire set of test statistics computed from the pilot data to estimate the effect size distribution, the power and the minimal sample size. Users first need to upload their pilot metabolomic data and perform the data processing and normalization steps as usual. Several diagnostic plots are then presented to allow users to check whether the test statistics follow an approximately normal distribution, and whether there are relatively a large number of *P* values that are close to zero (i.e. the effect indeed exists). When these assumptions are reasonably met, users can proceed to estimate the statistical power with regard to different sample sizes. The current implementation allows users to interactively explore the predicted powers for sample sizes ranging from 3 to 1000 samples per group. An example output from MetaboAnalyst's power calculations is shown in Figure 2D.

### Implementation

MetaboAnalyst 3.0 was implemented using the PrimeFaces 4.0 component library (http://primefaces.org/). The majority of the backend computations are carried out by R functions (www.r-project.org) based on the R programming language (v3.03). The application is currently hosted on a Linux server with 16G RAM and eight-core 2.6 GHz CPUs. For researchers who are routinely generating large volumes of metabolomic data or for those requiring secure data handling, MetaboAnalyst 3.0 is also available for download and local installation. Detailed installation instructions are available on the ‘Resources’ page. To further facilitate collaborative research and future development, all of MetaboAnalyst's source code is available as an Apache Maven project upon request. Due to our limited hardware resources, MetaboAnalyst currently offers only basic support for raw spectral data processing. Users are encouraged to use other dedicated and freely available tools such as *XCMSOnline* (30) and MZmine (31) for such tasks. The peak lists generated by these tools can be easily uploaded into our server for further downstream analysis.

## CONCLUSION

The development of MetaboAnalyst 3.0 has been driven primarily by user demands for new statistical methods to support emerging trends in metabolomics applications, by the demands for more efficient implementations and by requests for better data visualization to accommodate the tremendous increase in data analysis workloads. In re-designing and re-writing MetaboAnalyst 3.0, we directed a significant amount of effort and thought toward improving its computational efficiency. For instance, we made each module independent to reduce the large memory footprint and we significantly improved the efficiency in name mapping by adding several heuristic rules to the fuzzy search algorithm. We also looked for efficiencies in other areas. For instance, based on the Google Analytics and our own experience, we estimated that an average MetaboAnalyst analysis lasts for ∼20 min for new users. Therefore to maximize available computational resources and to reduce the load from zombie processes or abandoned analyses, we implemented a function to scan and kill processes that have run for more than 2.5 h to reclaim computational resources. We have also substantially increased the number (50X) of concurrent connections that are allowed on the application server. These procedures have dramatically increased the overall efficiency, performance and stability of the system. We are currently in the process of setting up a mirror site using the cloud service offered by Google Compute Engine.

In terms of overall scope and capabilities, MetaboAnalyst continues to be the most complete, freely available web-based resource for metabolomic data analysis. The main strengths of this web-based server are its user-friendly interface, its comprehensive data processing options, its wide array of univariate and multivariate statistical methods and its extensive data visualization and functional analysis support. In addition to these features, MetaboAnalyst 3.0 now offers a number of advanced statistical methods for multi-omic data analysis and clinical or translational research. Our intention is to continue to upgrade MetaboAnalyst over the coming years and to be as responsive to user requests as possible. Several areas of likely upcoming development include new modules for metabolite-wide association studies and environment-wide association studies as well as new modules for spectral analysis and compound identification.

## Supplementary Material

SUPPLEMENTARY DATA
