# Supplementary material for: MetaboAnalyst 3.0—making metabolomics more meaningful
Source: Nucleic Acids Res. 2015 Apr 20;43(Web Server issue):W251–7. doi: 10.1093/nar/gkv380 (PMC4489235; doi:10.1093/nar/gkv380)
Supplement: SUPPLEMENTARY DATA [file supp_43_W1_W251__index.html]

MetaboAnalyst 3.0—making metabolomics more meaningful — MetaboAnalyst 3.0—making metabolomics more meaningful — SUPPLEMENTARY DATA 

# MetaboAnalyst 3.0—making metabolomics more meaningful

## SUPPLEMENTARY DATA

**Files in this Data Supplement:**

- SUPPLEMENTARY DATA
