# Supplementary material for: MetaboAnalyst 3.0—making metabolomics more meaningful
Source: Nucleic Acids Res. 2015 Apr 20;43(Web Server issue):W251–7. doi: 10.1093/nar/gkv380 (PMC4489235; doi:10.1093/nar/gkv380)
Supplement: SUPPLEMENTARY DATA [file supp_gkv380_nar-00212-web-b-2015-File004.pdf]

Audience Overview

Mar 1, 2015 - Mar 31, 2015

All Sessions
100.00%

Overview

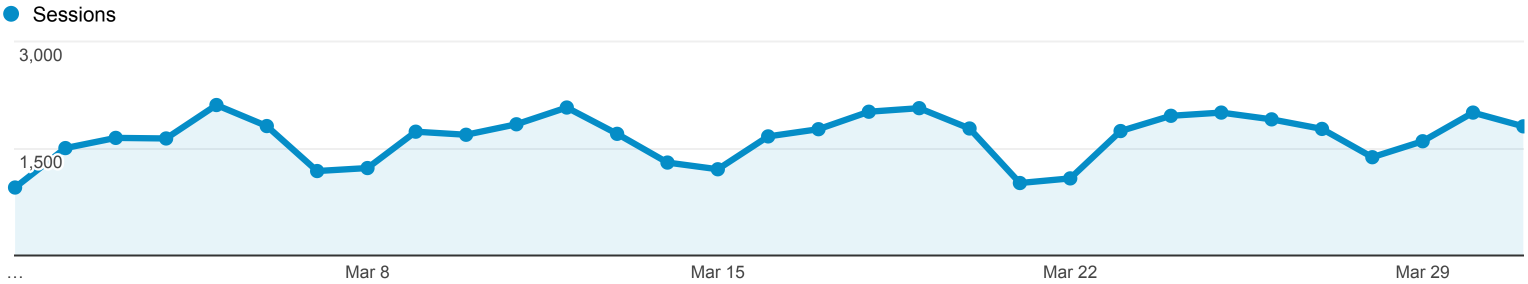

Sessions
51,203

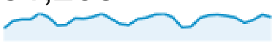

Users
3,229

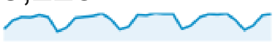

Pageviews
260,704

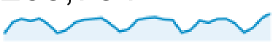

Pages / Session
5.09

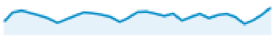

Avg. Session Duration
00:05:25

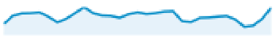

Bounce Rate
82.66%

% New Sessions
4.62%

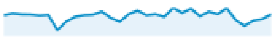

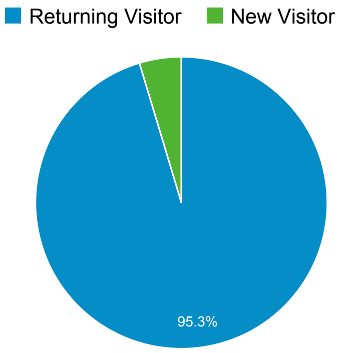

	Language	Sessions	% Sessions
1.	en-us	32,237	62.96%
2.	zh-cn	5,432	10.61%
3.	en-gb	2,113	4.13%
4.	de	1,866	3.64%
5.	ja	1,271	2.48%
6.	fr	1,236	2.41%
7.	pt-br	934	1.82%
8.	es	711	1.39%
9.	zh-tw	688	1.34%
10.	ko-kr	670	1.31%

© 2015 Google

Audience Overview

Oct 1, 2014 - Mar 31, 2015

All Sessions
100.00%

Overview

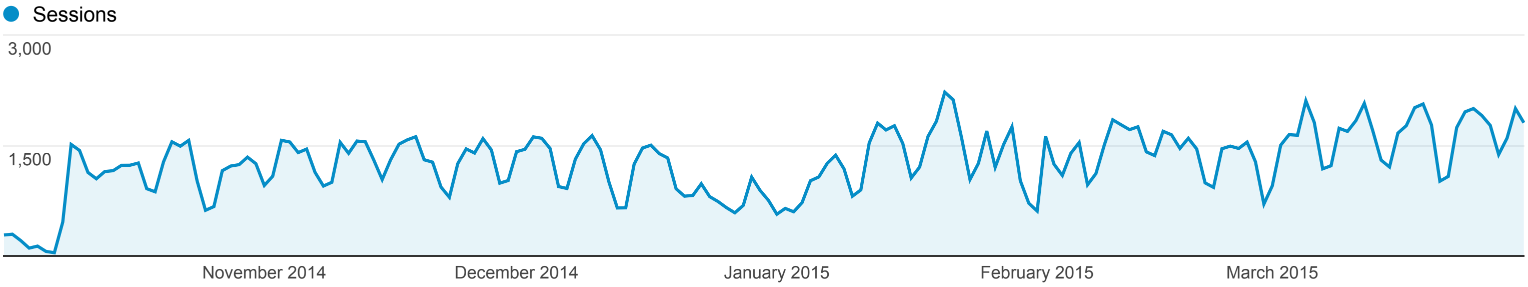

Sessions
232,440
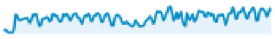

Users
12,061
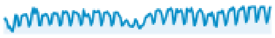

Pageviews
1,141,978
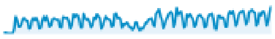

Pages / Session
4.91
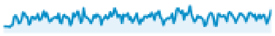

Avg. Session Duration
00:05:07
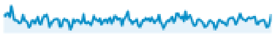

Bounce Rate
82.03%
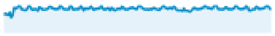

% New Sessions
4.77%
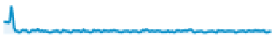

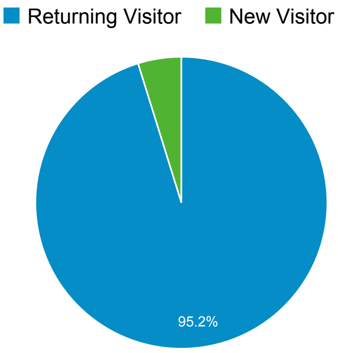

	Language	Sessions	% Sessions
1.	en-us	142,572	61.34%
2.	zh-cn	25,843	11.12%
3.	de	9,179	3.95%
4.	en-gb	8,678	3.73%
5.	pt-br	6,522	2.81%
6.	zh-tw	4,823	2.07%
7.	ko-kr	4,332	1.86%
8.	fr	3,883	1.67%
9.	ja	3,149	1.35%
10.	es	3,086	1.33%

© 2015 Google
